# Medical Transactions, Published by the College of Physicians in London. Vol. V

**Published:** 1816-08

**Authors:** 


*Medical Transactions, published by the College of Physicians in London. Vol. V.* 8*vo. pp.* 485. *London,* 1815.

The facility of intellectual production is, it should seem, like expedition in the mechanical arts, increased by practice. The four first volumes of the Medical Transactions were the work of more than half a century; while the fifth, equal in bulk, and perhaps superior in value, to its predecessors, has come to light after a lapse of only two years. A circumstance so unusual, at first excited within us serious apprehensions that the Royal College had suffered an abortion: but this fear is happily groundless, for on instituting a comparison between the younger production and its brethren, no extraordinary traces of weakness or immaturity are, we repeat, perceptible. We beg leave, therefore, to congratulate the learned and enlightened body on the increase of its prolific powers, so suddenly acquired; and sincerely wish that it may prove permanent as it was unexpected. We regret our inability to introduce the young stranger more early to our readers, with all the honours which its rank and parentage demand.

Thirty-five papers, unequal in interest, as various in subject and extent, compose the volume. We shall notice them in the order of their occurrence; and assign to each a space proportionate, not to the number of pages over which it is spread, but to its practical utility,—the sole criterion by which collections of this kind can be correctly judged. The largest bone contains not always the most marrow.

*I. Cases of Diabetes.* By Richard Patrick Satterley, M. D. &c.—Four cases of this affection are here detailed. The first and most interesting, is that of a male, aged 32. The symptoms, of six months duration, were most decidedly marked, and their first accession attributed by the man to drinking cold water when the body was overheated. Lumbar pains, severe and acute; thirst intense; bowels torpid; debility extreme. Sixteen quarts of sweet urine, with minute oily globules floating on its surface, were voided in twenty four hours. Each pint yielded, on evaporation, twelve drachms of "a black treacle." At seven different times, from February 19th, to March 11th. one hundred and twenty-six ounces of blood were drawn from the arm, with great and almost uninterrupted mitigation of all the symptoms, terminating in perfect recovery. The diet and other remedies prescribed, were animal food, lime-water, alum-whey; blister to the loins, purgatives, small doses of opium and compound ipecacuanha powder. The blood at first a black, treacle-like, homogeneous mass, gradually assumed a firmer consistence, and florid hue; and at last displayed the buff of inflammation. Even the immediate relief experienced from the operation was considerable, and made the patient anxious for a repetition of it more frequent than prudence would allow. The excretion of urine was progressively reduced to two quarts a day; but even then, although not only the general, but the *relative* quantity of saccharine extract was much diminished, some "sugar was found in it." About the close of March, convalescence was interrupted by an attack of pneumonia, attributable to no external cause, but resulting, we suspect, from that re-action of the system which the bold and judicious treatment of Dr. Satterley had excited. It readily yielded to "one or two additional bleedings." The patient is known to have remained well two years; and there is "reason to believe" that, at the date of the Doctor's communication, he had suffered no relapse. This we conceive to be a point of peculiar importance; for, according to our experience, few persons in whom suspension of diabetic symptoms has been effected by the tonic plan, have gone on four or five years without a recurrence of them, or invasion of some other malady, quite as dangerous and intractable. And, on the other hand, we are enabled to state, from the best authority, that so lately as the summer of 1814, no relapse had taken place in any of those patients, whose recovery from diabetes by a rigorous system of abstinence and depletion, Doctor Watt, of Glasgow, had six years previously announced.* In the other three cases recorded by Dr. Satterley, venæsection, although much less vigorously pushed, displayed an influence equally striking and beneficial. Two of these patients completely lost their diabetic symptoms under its employment; but the third, although signally relieved by a first abstraction of fourteen ounces of blood, would not again submit to the operation. The only important feature in Dr. Satterley's attempt to explain the cure of diabetes by remedies so widely differing in operation as opium and phlebotomy, is an opinion that the Sangrado system should be restricted to those cases, of which pain in the loins forms a conspicuous symptom. This, he is disposed to consider, indicates some inflammatory affection of the kidneys, and may "constitute a strong ground of difference in the treatment of the complaint." For our own parts, we have never seen a confirmed case of diabetes unattended by it. Recent observ-

---

* See his highly interesting "*Cases of Diabetes, Consumption, &c. with observations on the history and treatment of disease in general,* published by that gentleman in 1808.

ation leads us to believe that pure potash or soda, largely administered in combination with other medicinal or alimentary substances, would prove a powerful auxiliary to the lancet in *every* modification of diabetes mellitus. We cannot quit this paper without earnestly recommending its perusal to those gentlemen who find in that convenient word debility, a ready solution for every pathological problem; in the brandy-bottle, an unfailing panacea: and who, having erected an altar to a demon of their own creation, blindly heap it with—human sacrifices.

*II. On Leucorrhœa.* By John Latham, M.D. F.R.S. &c.—From this paper of the President's, occupying sixteen pages, we can extract but little. In all cases of obstinate leucorrhœa, he very properly insists on the necessity of careful and reiterated examination *per vaginam*, in order to ascertain correctly its source. When the disease has been induced by "detention of a small portion of the menstrual flux within the vagina" and its subsequent decomposition, or by accumulation of sordes within the labia, simple injections of warm water will commonly suffice to remove it. Fomentations, abstractions of blood, local and general, and blisters, are indicated, if it be attributable to any slow inflammatory action established within the vagina; and, afterwards, tonics and astringents, to obviate the consequent relaxation. At the next menstrual period, moreover, all irritative action should be quieted by rest, opiates, and topical application of heat. But the doctor's main object is evidently to recommend injections of the "*liquor plumbi acetatis dilutus*" in these cases; from the use of which "just warm, and gradually increased to about double its common power, he has obtained, even under circumstances apparently hopeless, the most happy results. If it be really capable of mitigating the pains, and checking the progress of uterine cancer, we shall be among the first to hail it as a "fortunate discovery." It is somewhat singular that the learned president no where mentions the meloe vesicatorius as a remedy in leucorrhœa. We have administered it with signal benefit in several cases which had previously resisted all the common resources of medicine. Persons, in whom the flux is complicated with symptoms of impending phthisis, are, according to our experience, the most conspicuously relieved by its employment.*

* To Dr. Roberton, we believe, is due the credit of having first ushered into general notice the use of the blistering fly in these affections.

*III. Some account of a contrivance which was found of singular benefit in stopping the Excoriation and Ulceration consequent upon continued pressure in bed.* By W. HEBERDEN, M. D. F. R. S. &c.—This consists of a frame, about six inches in depth, and of such dimensions as to fit the bedstead of the person for whose use it is designed. In the centre of it, is a drawer so constructed, as to admit of being drawn out on either side, containing a pan which, when properly adjusted, is immediately below a circular opening in the roof of the drawer, and thus receives what ever may be discharged from the patient's body. The mattrass, the under sheets, and blankets, have each a circular hole cut in them, corresponding to the situation of the pan. A circular cushion surrounds the orifice of the mattrass; and, for the purpose of cleanliness, a piece of oiled silk covers its margin. The frame has a desk at the upper part, whereby the body of the patient may be elevated; and the mattrass has a division in it corresponding to the base of the desk; so that the higher part may accommodate itself to the necessary elevation, without disturbing the remainder. A foot board, Dr Heberden suggests, would be an useful addition to the frame, as the patient would thus be prevented from sinking too forward on the hole, when, from continued pressure, the mattrass has given way. In describing this apparatus, some allusion should, we think, have been made to the masterly contrivance of Sir James Earle, destined for the use of the helpless and infirm; to which, however inferior in construction, it bears a strong resemblance in principle, and from which, in fact, the idea of it was evidently borrowed.*

*IV. On Colica Pictonum.* By EDWARD ROBERTS, M.D. F. R. S. &c. — The object of this paper is to shew the efficacy of nitrate of silver in the removal of those terrible and distressing symptoms, which frequently result from exposure to saturnine emanations. In the two cases, which are detailed in illustration of its successful employment, the remedy was administered in doses of from one to five grains, three or four times a day, and was preceded

---

Our evidence respecting its operation is most decidedly favourable; and we gladly do justice to the medicine, whatever we may think of the man. The public and indignant manner in which Dr. Baillie has recently resented his disgraceful conduct, is well known. REV.

* See Sir James Earle's "letter containing some observations on Fractures of the lower limbs," &c. 1807.

by a draught of castor oil. Combination with opium was found necessary in the last case, from the activity of its operation on the bowels. Dr. Roberts thinks, that in these cases, the nitrate of silver exerts an influence both on the nervous system and the bowels; and removes the disease "probably by the same mode of action as it frequently alleviates epilepsy and cures chorea." In point of composition, this paper is exceedingly defective.

*V. Some Observations respecting the Medicines usually given in Worm Cases, with remarks upon the collateral advantages sometimes derived from them in Cases of Epilepsy.* By John Latham, M. D. &c.—An indifferent performance truly; containing, first, some common place observations on the efficacy of spirit of turpentine in expelling intestinal worms; secondly, a suggestion, that in order to insure their successful operation, all vermifuge remedies should be exhibited in doses sufficiently large for their "distribution throughout the whole intestinal canal"; and lastly, some brief remarks on the employment of turpentine in epilepsy. Both the President and Dr. Percival of Dublin, it seems, have administered it with occasional success against the epileptic paroxysm; but the Dublin physician sets the mind of Dr. Latham at ease respecting the "honour of priority" in its recommendation, by most handsomely acknowledging "that he first employed this remedy from the recommendation which I had given of it in my book on Diabetes above mentioned." Dr. Latham very properly adds, that no good can be expected from the use of turpentine in cases of epilepsy connected with diseased organization of the brain: indeed, under such circumstances, its administration would, we conceive, serve only to aggravate the malady by exciting that activity in the vessels of the morbid organ, which all the efforts of the physician should be directed to tranquillise and arrest. We confidently predict, that in epilepsy originating from deranged function of the nervous system, the *melöe vesicatorius,* proposed by Dr. Lara,* of Portsmouth, will be found a much more powerful remedy than the spirit of turpentine. At least our comparative trials of both the substances in question warrant this conclusion.

---

* See the "New Medical and Physical Journal," Vol. IX. page 201. We beg leave to remind Dr. Lara, that the pledge there given to the public, yet lies unredeemed. Rev.

*VI. On Cachexia Aphthosa.* By the same.—This is the *aphthoides chronica*, of Hillary; a disease frequently occurring in warm climates; rarely in our own. It is dependent on a "cachectic state of the whole system," characterized by ulceration of the mouth, tongue, fauces, and intestinal canal. Seldom cured, it often admits of palliation by the resources of medicine. We will attempt, from the President's rambling description, to trace a succinct, but clear history of this formidable affection. Dyspepsia, with acidity, eructation, heart-burn, signalizes its commencement. No fever: no pain or enlargement in the region of the liver: no jaundice. The stools indicate a defective biliary secretion; and thus confirm the inference drawn from the "olive-like complexion, that diseased action prevails in the liver. This organ is *believed* to diminish in volume as the disease advances. Slow hectic fever, with a weak and somewhat accelerated pulse, at length, shews itself: urine scanty, of a milky or whey-like hue: pimples on the margin of the tongue, with superficial blisters in the buccal cavity, and heat and soreness in the epigastric region. Diarrhœa, consequent on extension of the morbid action to the whole intestinal tube, supervenes. The excrement more strongly resembles "thick oatmeal gruel in an incipient state of fermentation than fæces." It is sometimes sour, rarely offensive, except when deriving from occasional admixture with blood or mucus, "the peculiar odour of putrid animal matter." The paroxysm, here delineated, sometimes exhibits a remission spontaneous or artificial; but speedily recurs in aggravated characters: and the mind of the patient becomes infected with gloomy apprehensions.

"The ulceration of the tongue and mouth then grows more painful; the salivary discharge is, every moment, provoked from a denuded surface, and the disordered state of the stomach and intestines is intolerably oppressive. Death, in its approach, however, still unkindly lingers, and seems, as it were, unwilling to overtake its languid victim, until, worn down with inquietude and fatigue, he sinks into a state of exhausted apathy, and life deserts him."

The skin, we should have observed, is commonly rough and dry. Dr. Latham has frequently traced the origin of the disease, when occurring in this country, to indulgence in vinous and spirituous liquors. He thinks that it possesses some resemblance to scurvy, and may be referred to imperfect chylification from the deficiency of the biliary stimulus. He has also seen occasionally a state of the system not unlike that "on which *cachexia aphthosa* depends, produced by (we would rather say, producing) chronic dysentery." Now we will venture to affirm, from our own observation, that the analogy is, in the latter case, by far the most striking and correct; and our inference will presently be found to derive additional stability from the treatment by which the aphthous affection is most successfully combatted. In its first, or simply dyspeptic stage, the disease may be often cured by a few doses of calomel, and bitter infusion combined with rhubarb or magnesia, especially if it occur in a temperate climate. But when engendered beneath the influence of a tropical sun, or arrived at a more advanced period, it will not yield thus readily. Here, Dr. Latham very judiciously recommends mercury, as an external application in the form of unguent or plaster to the hepatic region; internally, in that of submuriate, or grey oxyd, combined with opium; repeated emetics of ipecacuanha; infusions of cascarilla or cinnamon bark, of lemon or pomegranate rind, in lime-water; occasional aperients of rhubarb or magnesia: lime-water and milk for the beverage; London bread, as containing alum, and all light and nutritious alimentary substances, for solid food; and, lastly, utter abstinence from wine, spirits, and, we would beg leave to add, from all fermented or fermenting liquids. As topical applications to the mouth and throat, compounds of barley-water, mucilages, syrups, with borax or alum, will be most productive of relief; and, with the same view, Dr. Latham has successfully prescribed a few grains of lapis calaminaris, or compound powder of ipecacuanha, in almond emulsion or other mucilaginous draught. But is it not passing strange that the Doctor, without any comment or precaution, suggested by its deleterious properties, should strongly extol the employment of his favourite "*liquor plumbi acetatis dilutus*," both as a gargle and injection, in this malady? Ablution of an ulcerated throat or rectum with an active preparation of lead can scarcely, as a mere palliative, be productive of advantages, sufficient to compensate for the risk of its introduction into the system from these cavities. At all events, the practitioner, who, tempted by such powerful recommendation, may decide upon its use, will do well to recollect, that the *soluble sulphates constitute* the best antidote to the poison of lead. Opium is the remedy principally to be relied on in mitigating the pain, exhaustion, and despondency, which signalize the ravages of the disease towards its fatal termination. In two cases of this nature, which, within the last year, have fallen under our observation, mercury, with infusion of cinchona in lime-water, a an internal remedy, and solution of borax, as a gargle, were exhibited with great success; but in one of our subjects, a middle-aged woman, "fond of good living," the affection did not completely yield, until the gums had, for several days, displayed the influence of the mercury upon the system. It is somewhat singular, that this plan of treatment was suggested to us by views of the pathology of the aphthous affection, precisely resembling those upon which the practice of the learned President is founded; at a period when the contents of this excellent paper were necessarily unknown to us.

*VII. Observations upon some Cases of Paralytic Affection.* By Richard Powell, M. D. &c.—The malady, to which Dr. Powell here solicits our notice, is "a paralytic condition of the nerves of particular parts induced by the operation of cold." It frequently attacks one side of the face after exposure to severe cold; and assumes, from the consequent loss of muscular power, the aspect of hemiplegia. The topical application of heat and moisture, and the excitement of perspiration by powerful sudorifics, are commonly adequate to its removal. Might not friction, electricity, or galvanism, be employed with success in obstinate cases? The following example of universal invasion of *paralysis a frigore,* we shall record, as well illustrating the affection, and shewing its independence on any organic lesion of the nervous system.

"A watchman, on quitting his duty after a night of severe cold, was attacked by sudden and violent general pains in his limbs; which soon departed and left him in a state of universal palsy of the muscles of voluntary motion. He had lost all command over the muscles of his limbs or trunk; but the joints were unaltered in their external appearance: they were perfectly flexible, and it gave no pain, if you moved them in any direction. The sphincters also of the rectum and bladder had lost their usual powers of retention, and he passed both stools and urine involuntarily and unconsciously. His circulation was not affected in any noticeable degree, and his mind retained its usual powers. His voice was not lost. I attempted the relief of this unfortunate man by the hot bath and a variety of remedies without success. He died, and after death a very careful examination of the brain was made. No congestion, no effusion, no alteration of structure of any kind, was discoverable in it."

*VIII. On the Effects of certain Articles of Food, especially Oysters, on Women after Child-birth.* By JOHN CLARKE, M. D. &c.—Six cases are here recorded, in which the ingestion of oysters by puerperal females, brought on a train of the most violent and terrific symptoms, characterizing inflammation of the brain. Three of the cases terminated in fatal effusion. The other three patients probably were rescued from a similar fate, by an early and vigorous plan of depletion.

"The conclusion to be drawn from the consideration of the cases which have been related above, is, that the state of pregnancy not only induces such a flow of blood to the head as to dispose it to be violently affected by the strong exertions of labour, so as to induce puerperal convulsions; but also to render it liable to be particularly acted upon, for some time after child-birth, by sympathy with the stomach, when indigestible substances, especially the fishes of the bivalve class, have been eaten."

Hence it follows, that medical practitioners should, on all occasions, warn their female patients to abstain rigorously from oysters for some time after delivery; and that, when the alarming symptoms, consequent on their employment, have been developed, an early and active exhibition of all the remedies indicated against cerebral inflammation will be necessary to avert impending destruction. We should, ourselves, lay particular stress upon copious bleeding from the jugular vein. Some unsound notions in physiology shade the excellencies, without diminishing the practical value of this paper. But, in reviewing one of the latest productions of an estimable writer and physician, criticism forgoes its wonted asperity for the kindlier emotions of reverence and regret: and a passing eulogy on the talents of Dr. Clarke is more congenial to our feelings than the language of correction.

*IX. Upon a Case of Stricture of Rectum, produced by a Spasmodic Contraction of the internal and external Sphincter of the Anus.* By MATHEW BAILLIE, M. D. F. R. S. &c. &c.—We cannot, perhaps, give a more correct idea of this species of stricture than by sketching a sort of parallel between it and that of more common occurrence, and intractable character. *Common stricture of the rectum,* generally situated two or three inches above the external sphincter; a hard irregular ulcer frequently found in the coats of the bowel, at the seat of stricture; that portion of the intestine between the stricture and anus, possessing its natural organization and capacity, attended with great constitutional disorder, and terminating in weakness, emaciation, and death. Generally incurable.

*Spasmodic contraction of the sphincters.*—The stricture, seated at the anus, and not implicating the cavity of the bowel above; the membrane of the rectum, soft and natural; the general health unimpaired. The prognosis favourable. In both cases, the fæces are flattened, small, and somewhat serpentine or twisted. The spasmodic contraction will probably yield to the use of the bougie. Sometimes, it wears off spontaneously. Dr. Baillie describes it as a rare occurrence.

*X. Some Observations respecting the Green Jaundice.* By the same.—The green jaundice is much less commonly observed than the yellow, and widely differs from it in many other respects, besides that of colour. It rarely, though sometimes, attacks young persons; men more frequently than women; and seems less connected with intemperance than the other species. The liver of those affected by it is often enlarged, hard, and tuberculated throughout, sometimes partially. There is little pain in the region of this organ; often tenderness on pressure. Peritonéal dropsy is not a common consequence of it. The pulse is, for the most part, natural: the stools generally pale, sometimes tinged with bile; the urine much loaded with bile, and staining linen, though not always in slighter cases, of a deep yellow colour: it is often less scanty than in the other variety of jaundice, generally clear, and seldom deposits a pink sediment. There is sometimes a distressful itching of the skin, and strong sensation of heat in the hollow of the hands and feet. Appetite and digestion are, in some cases, much impaired. The patient very rarely recovers from this malady, which, though slow in progress, is almost constantly fatal in termination. Marasmus and debility mark its eventful close. Even where the issue is fortunate, the green colour, although occasionally varying in depth of shade, never completely forsakes the skin. Mercury may relieve, for a time, the uneasy sensations of the patient in this malady; but never operates a permanent cure. Neutral salts have been daily exhibited with some advantage. Some essential difference probably exists between the bile of yellow, and that of green jaundice; and it is the opinion of Dr. Baillie, that accurate chemical analysis of the latter, while elucidating this difference, might, perhaps, also "suggest some improvement in the mode of treating the green jaundice." It is a deeper shade of this malady to which the designation of black jaundice* has frequently been applied.

*XI. Some Account of a Rash liable to be mistaken for Scarlatina.* By William George Maton, M. D. F. R.S. &c.—This eruption strongly resembles *scarlatina simplex* in its obvious characters; and is, like that, propagated by infection, but differs from it in the appearance of the tongue, which is slightly furred; in the longer period which elapses between the exposure to the infection and manifestation of the rash; and in the non-occurrence of desquamation of the cuticle afterwards. Like *roseola* and *urticaria,* it is attended with itching or tingling of the skin; but, in its universal diffusion and infectious character, the analogy ceases. The constitutional derangement ushering it in is usually very slight, and speedily yields to mercurial purgatives. In some cases, observed by Dr. Maton, the decline of the efflorescence was marked by the developement of small "tumors or tuberosities," about the integuments of the occiput and nucha. They were variable in size, flattened, irregular in circumference, sore, at times painful, and some of them slightly discoloured. They continued some weeks. The menses were interrupted from the commencement of the eruption, and long after its decline, in two instances. Dr. Maton, perhaps, attaches to his "non-descript" a degree of importance greater than it will acquire in the estimation of those whom practice has made most familiar with the unsteady and complicated phænomena of cutaneous diseases: at least, until reiterated and severe observation shall have established, beyond the possibility of illusion, its distinct character, and shewn that it is not a variety of *scarlatina simplex,* modified and diverted from its ordinary route, by the operation of some unknown but peculiar causes.

*XII. Some Observations on a particular Species of Purging.* By Matthew Baillie, M. D. &c.—The complaint here delineated is of a most formidable nature,

---

* Our English term *Jaundice,* being derived from the French Jaune, *yellow,* the appellation of *green* or *black* Jaundice involves an obvious absurdity.—Rev.

rarely cured, though sometimes admitting of palliation. It consists, when very violent, of abundant and numerous evacuations of pale fæces, resembling a mixture of lime and water, frothy on the surface, often smelling very sour. The patient has usually a sallow countenance; is thin, but not much emaciated. The appetite is sometimes impaired; the pulse natural or a little accelerated. A white fur covers the tongue, but the mouth displays neither soreness nor aphthæ. The urine is more deeply-coloured than natural, generally clear, sometimes turbid. The abdomen presents no tumor or soreness, on pressure; but the bowels are often distended by flatus. Dissection has thrown no light on the source of this incurable malady. Rarely occurring in this island, its attacks are principally confined to those whose constitutions have suffered from long residence in a warm climate, and from liver diseases; and, probably, from this cause, men are more frequently its victims than women. There is a milder form of the complaint, in which the excrement, though pale and sometimes variously coloured, has the consistence of pudding, and is less frequently voided. It sometimes even becomes figured, assumes a darker colour, and approaches are made towards recovery, but the improved state is never permanent; and under this alternate succession of amendment and relapse, the destined victim sometimes lingers several years, till, at last, he sinks exhausted. Relief is sometimes obtained by the administration of very small doses of mercury, and of bitters combined with opium. A diet, consisting almost entirely of rice, has also been prescribed with advantage. Mental anxiety and perturbation accelerate the progress of this uncontrolable malady. The present paper, like the two former productions of the same celebrated writer, which enrich this volume, bears evident traces of a master's hand.

*XIII. A Case of Water in the Head, unattended by its usual Symptoms, with Observations.* By W. HEBERDEN, M.D. F.R.S. &c.—We beg leave, in the first place, to transcribe the correct and masterly description of the morbid appearances, observed in this case; for which Dr. Heberden is indebted to Mr. Earle. It not only constitutes the most valuable feature of the communication; but may serve as a model of the concise and luminous, in morbid anatomical description.

"On attempting to remove the calvaria, the dura mater adhered with so much firmness as to split into layers rather than quit its connection with the bone. The arachnoid membrane was thickened, of a milky colour, and much elevated by the subjacent fluid, which pervaded the cellular structure of the pia mater to the amount of four ounces. The substance of the brain was healthy. The ventricles were greatly enlarged, and contained about eight ounces of transparent water. The plexus choroides was pale and void of vessels, containing many minute vesicles resembling hydatids, and one solid tumor, which appeared to contain the same calculous matter, which is met with in the pineal gland. The internal carotid and basilary arteries, with many of their primary branches, were ossified. In the chest, the lungs were found firmly adhering to the parietes; and some small tumors, containing calculous matter, probably phosphate of lime, were dispersed through their substance. In the heart, the valves of the left auriculo-ventricular opening were partially ossified; those of the aorta completely so, and small depositions of bony matter were found in the tendinous portions of the carneæ columnæ. The coronary artery was ossified through its whole extent. The aorta, soon after emerging from the ventricle, formed a true aneurismal pouch, consisting of a dilatation of the coats of the artery, without any rupture of its internal lining. The descending thoracic and abdominal aorta, with all their primary branches, were converted into cylinders of bone, as also were the external and internal iliacs. The intestines were, in many places, firmly adhering by their peritoneal surfaces, apparently the result of former attacks of inflammation. At each abdominal ring there was a small hernial sac. The liver was healthy: the spleen covered with a bony deposition between its peritonæal covering and tunica propria. The bladder contained some turbid urine, and a small spot near its neck had a blackened appearance. The prostate gland was enlarged. The kidnies were healthy. In the left testicle, there was a collection of water."

The subject of this interesting dissection was aged about eighty. He had been many years deaf, was of sound constitution, but had occasionally suffered attacks of fever, with expectoration; and once or twice giddiness. About six weeks previously to his decease, he was supposed to have had a fit during the night, as he had fallen from his bed, and seemed confused and weak. His recollection was soon restored; but the pulse was quick, and the appetite gone. He had neither pain nor giddiness. In a short time, he regained his usual state: "his appetite had returned; his pulse was equal and natural; his breath perfectly free; his voice strong, and his understanding and memory clear and good." But, soon afterwards, at the close of a day of seeming "indisposition," his physician found him senseless, with a pulse almost imperceptible; evidently dying. He expired before morning. The paper is closed by some common observations on the wonderful power possessed by the living system, of "accommodating itself to new circumstances;" and by the quotation of an analogous case from Morgagni:—both of which are valuable—only from the space they serve to occupy.

*XIV. Case of Chorea in an aged Person, cured by Musk.* By W. G. MATON, M. D.—The patient was a Lady, of infirm irritable habit, and fretful disposition; aged seventy. The disease was strongly marked; and the efficacy of musk, prescribed in doses of ten grains every six hours, prompt and conspicuous. Almost all the other articles of the materia medica, indicated in such cases, had been previously tried without success. We are, however, surprised that Dr. Maton should not consider *furred tongue* and *torpid bowels,* as indications of "fault" in the "organs of digestion."

*XV. A Case of natural Small-Pox, occurring several Years after Inoculation with Variolous Matter, in consequence of the Progress of the inoculated Pustules being interrupted.* By Mr. MILLINGTON.

> A Barber's block mid busts of Bronze;—

> A *Goose* among the College *Swans.*

Exhausted by this sublime poetic flight, we shall repose awhile, and resume the analysis in our next.

---

